# Eosinopenia is a reliable marker of sepsis on admission to medical intensive care units

**DOI:** 10.1186/cc6883

**Published:** 2008-04-24

**Authors:** Khalid Abidi, Ibtissam Khoudri, Jihane Belayachi, Naoufel Madani, Aicha Zekraoui, Amine Ali Zeggwagh, Redouane Abouqal

**Affiliations:** 1 Medical Intensive Care Unit, Ibn Sina University Hospital, 10000, Rabat, Morocco; 2 Laboratory of Biostatistics, Clincial and Epidemiological Research, Faculté de Médecine et Pharmacie - Université Mohamed V, 10000, Rabat, Morocco

## Abstract

**Introduction:**

Eosinopenia is a cheap and forgotten marker of acute infection that has not been evaluated previously in intensive care units (ICUs). The aim of the present study was to test the value of eosinopenia in the diagnosis of sepsis in patients admitted to ICUs.

**Methods:**

A prospective study of consecutive adult patients admitted to a 12-bed medical ICU was performed. Eosinophils were measured at ICU admission. Two intensivists blinded to the eosinophils classified patients as negative or with systemic inflammatory response syndrome (SIRS), sepsis, severe sepsis, or septic shock.

**Results:**

A total of 177 patients were enrolled. In discriminating noninfected (negative + SIRS) and infected (sepsis + severe sepsis + septic shock) groups, the area under the receiver operating characteristic curve was 0.89 (95% confidence interval (CI), 0.83 to 0.94). Eosinophils at <50 cells/mm^3 ^yielded a sensitivity of 80% (95% CI, 71% to 86%), a specificity of 91% (95% CI, 79% to 96%), a positive likelihood ratio of 9.12 (95% CI, 3.9 to 21), and a negative likelihood ratio of 0.21(95% CI, 0.15 to 0.31). In discriminating SIRS and infected groups, the area under the receiver operating characteristic curve was 0.84 (95% CI, 0.74 to 0.94). Eosinophils at <40 cells/mm^3 ^yielded a sensitivity of 80% (95% CI, 71% to 86%), a specificity of 80% (95% CI, 55% to 93%), a positive likelihood ratio of 4 (95% CI, 1.65 to 9.65), and a negative likelihood ratio of 0.25 (95% CI, 0.17 to 0.36).

**Conclusion:**

Eosinopenia is a good diagnostic marker in distinguishing between noninfection and infection, but is a moderate marker in discriminating between SIRS and infection in newly admitted critically ill patients. Eosinopenia may become a helpful clinical tool in ICU practices.

## Introduction

Sepsis is one of the most common causes of morbidity and mortality in the intensive care unit (ICU) [1]. Sepsis is generally characterized by clinical and laboratory parameters that are not specific and can mislead because these parameters often change in critically ill patients with systemic inflammatory response syndrome (SIRS) [2].

Sepsis and noninfectious SIRS produce very similar clinical features. It is very important that clinicians have the tools to recognize and diagnose sepsis promptly because early diagnosis and treatment may lead to improvement in both mortality and morbidity [3]. An early diagnosis of sepsis before receiving the results of microbial culture would certainly facilitate the choice of antibiotic therapy and reduce the patient mortality. Unfortunately, the availability of a highly specific sensitive marker of infection is still not satisfied [4]. An ideal marker of infection would be highly specific, highly sensitive, easy to measure, rapid, inexpensive, and correlated with the severity and prognosis of infection. Recent studies have suggested an important role of procalcitonin plasma concentration monitoring [3-12], and more recently the triggering receptor expressed on myeloid cells 1 [13], in the clinical diagnosis of sepsis, because they differentiate sepsis from noninfection causes of SIRS. The use of procalcitonin in developing countries such as Morocco, however, remains very expensive and hardly accessible in ICUs.

It is already known that eosinopenia typically accompanies the response to acute infection [14]. This marked reduction in the number of circulating eosinophil leucocytes in acute infection was first described by Zappert in 1893 [15], and was utilized during the first quarter of the last century as a useful diagnostic sign [16]. After the observation that eosinopenia is part of the normal response to stress [17], it was assumed that eosinopenia of acute infection is a secondary response to stress caused by the infection [18].

The value of this old marker of acute infection was tested by Gil and colleagues [19]. To our knowledge, however, there is no earlier study testing the value of eosinopenia in the diagnosis of sepsis in the ICU. This is the first report of the reproduction of eosinopenia in acute infection on ICU admission.

The aim of the present study was to assess the value of eosinopenia in differentiating sepsis-related conditions (sepsis, severe sepsis, septic shock) from other noninfection causes of SIRS in Moroccan critically ill patients on ICU admission.

## Materials and methods

### Study design and setting

A prospective study was performed of all patients consecutively admitted to a 12-bed medical ICU of Rabat University Hospital between February and May 2006. Patients who died or were discharged within 24 hours after admission were excluded from the study. Rabat University Hospital is the referral venue for habitants in Western-North Morocco. The 12-bed medical ICU admits approximately 550 patients annually with an average age of 40 years. Surgery patients, coronary patients, neonates and burn patients are treated in specialized units. The study protocol was approved by the hospital ethics committee. Informed consent was not demanded because this observational study did not require any deviation from routine medical practice.

### Data collection and definitions

At the time of ICU admission, for each patient we evaluated their age, gender, principal diagnosis, and vital signs (body temperature, heart rate, respiratory rate, systolic and diastolic arterial pressure, and urine rate). The Mc Cabe index [20], the Acute Physiology and Chronic Health Evaluation II score [21] and the Sequential Organ Failure Assessment score [22] were also recorded on admission. The white blood cell count, the eosinophil cell count and the C-reactive protein (CRP) level were only systematically recorded on admission to the ICU and not daily during the entire ICU stay.

Blood samples were obtained by venipuncture on admission, and subsequently each morning at 07:00 hours. The clinical practice in the unit follows the recommendations of the task force of the American College of Critical Care Medicine of the Society of Critical Care Medicine [23]: blood cultures were taken if a patient's body temperature exceeded 38.3°C, if a patient had clinical signs of severe sepsis, or if there was a need for vasopressor therapy for suspected septic shock. The samples for blood cultures were taken from two different sites, most commonly through intravascular devices (arterial cannula, central vein catheter or pulmonary arterial catheter.

Other cultures including urine, cerebrospinal fluid, and respiratory secretions were obtained according to the clinical circumstance and before antibiotics were given. Empirical antibiotic treatment was based on the presumptive diagnosis and received on the day of bacteriological cultures. When bacteriological results became available, the antibiotics were changed according to the pathogen isolated and the antimicrobial susceptibility test results.

According to the Criteria of the American College of Chest Physicians/Society of Critical Care Medicine [2], patients were classified as having SIRS, sepsis, severe sepsis, or septic shock at the time of admission. SIRS is defined by two or more of the following criteria: body temperature >38°C or <36°C, heart rate >90 beats/min, respiratory rate >20/min or PaCO_2 _< 32 Torr, and white blood cell count >12,000 cells/mm^3^, <4,000 cells/mm^3^, or >10% immature forms. Sepsis is a SIRS associated with the presence of an infectious process. Severe sepsis is a sepsis associated with organ dysfunction, hypoperfusion, or hypotension (systolic blood pressure <90 mmHg or a reduction ≥ 40 mmHg from baseline). Septic shock is a subset of severe sepsis and is defined as a persisting sepsis-induced hypotension despite adequate fluid resuscitation.

Infection was diagnosed by textbook standard criteria [24] and was categorized according to the following: culture\microscopy of a pathogen from a clinical focus; positive urine dip test in the presence of dysuria symptoms; clinical lower respiratory tract symptoms and radiographic pulmonary abnormalities that are at least segmental and not due to pre-existing or other known causes; infection documented with another imaging technique; lumbar puncture when meningitis was suspected; obvious clinical infection (erysipelas); and identification of a pathogen by serology or by PCR.

Importantly, two investigators retrospectively reviewed all medical records pertaining to each patient and independently classified the diagnosis as SIRS, sepsis, severe sepsis, or septic shock at the time of admission on the basis of the review of the complete patient charts, results of microbiologic cultures, and radiographs. Both intensivists were blinded to the eosinophil cell count and CRP levels. Concordance among the two independent investigators was excellent and the reliability was high (κ = 0.94).

We assessed the value of eosinopenia as marker of sepsis by comparing the eosinophil cell count between noninfected patients (negative, SIRS) and infected patients (sepsis, severe sepsis, and septic shock), and between SIRS patients and infected patients on the day of admission to the ICU.

### Laboratory measurement

Blood samples were collected in microtubes containing ethylenediamine tetraacetic acid anticoagulant. The white blood cell count and the eosinophil cell count were performed by the Coulter (Gen·S) hematology analyzer (Beckman Coulter, Fullerton, CA, USA). To determine the CRP level, blood samples were drawn into green-top vacutainer tubes containing lithium-heparin as anticoagulant. Plasma CRP was also measured by immunoturbidimetry using the analyzer Cobas Integra (Roche Diagnostics, Mannheim, Germany). The limits of detection were 0.071 mg/dl.

### Statistical analyses

Data are presented as the mean ± standard deviation for variables with a normal distribution, and as the median and interquartile range for variables with skewed distributions. Parametric or nonparametric tests were used for continuous variables as appropriate after the normality of the distribution was tested by the Kolmogorov-Smirnov test with Lilliefors correction. Statistical differences between groups were evaluated by the chi-square test for categorical variables. Comparison of group differences for continuous variables was carried out by one-way analysis of variance or the Kruskal-Wallis test. Bonferroni's *post hoc *test was used to locate the significance. The Spearman rank correlation coefficient (*r*) was calculated to describe the quantitative relationships between the eosinophil count and clinical or biological features.

The best cutoff value was chosen using Younden's index. Receiver operating characteristic curves and the respective areas under the curves were calculated for eosinophils and CRP. The sensitivity, specificity, and positive and negative likelihood ratios (with 95% confidence intervals (CIs)) were calculated at the best cutoff value. A multiple logistic regression was performed to explore the association between the eosinophil cell count, CRP levels, and infection, controlling for the potential confounders (age, Acute Physiology and Chronic Health Evaluation II score, Mc Cabe index, and Sequential Organ Failure Assessment score). Results are presented as the odds ratio and 95% CI.

A two-tailed *P *value <0.05 was considered significant. Statistical analyses were carried out using SPSS for Windows, version 13.0 (SPSS, Inc., Chicago, IL, USA).

## Results

### Characteristics of the study sample

**Figure 1:**
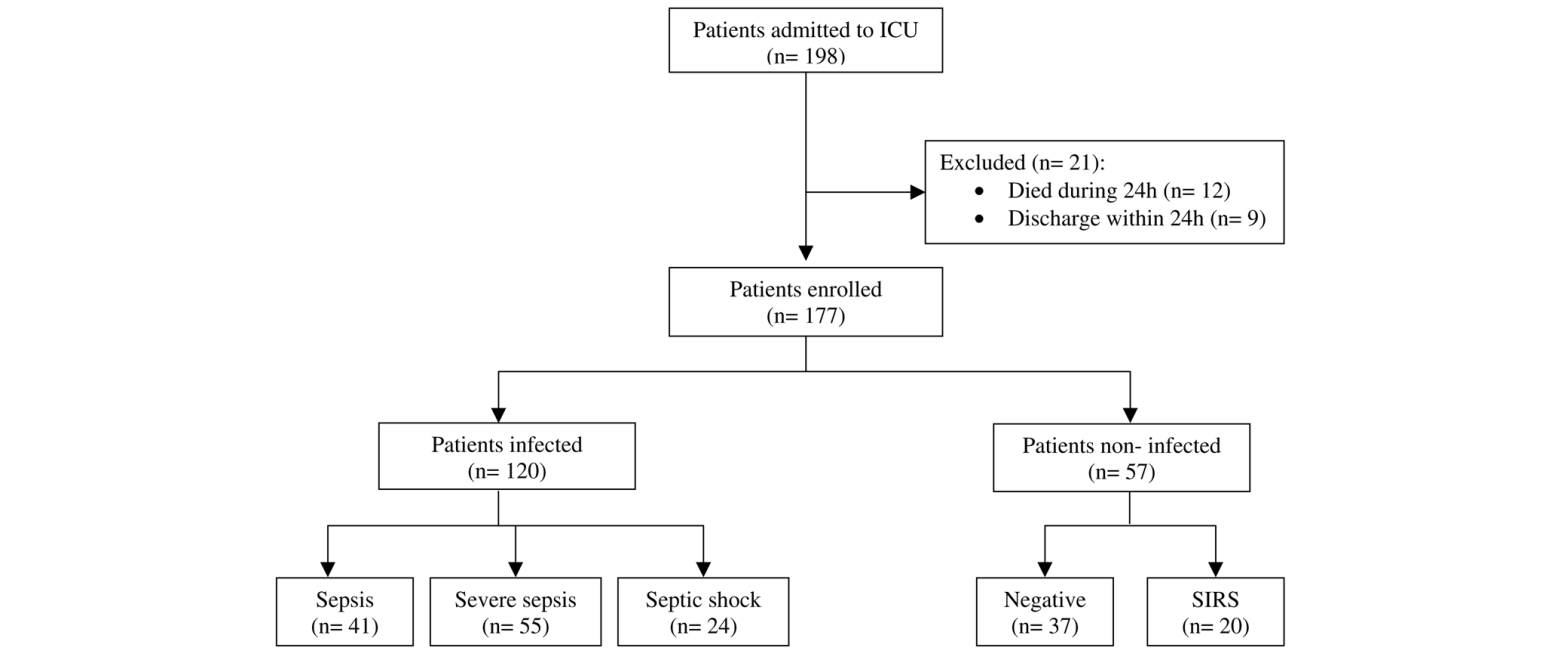
Patients included and excluded from the study. ICU, intensive care unit; SIRS, systemic inflammatory response syndrome.

During the study period, 198 patients were admitted to the ICU (Figure 1), and 21 patients were excluded because of death (n = 12) or discharge within 24 hours (n = 9). The remaining 177 patients were enrolled into the study, having a mean age of 42 ± 19 years. Mortality during the ICU stay occurred in 58 out of 177 patients (33%). At the time of admission, 120/177 patients (68%) had an infection. The sites of infections and clinical characteristics of the study patients are presented in Table 1.

**Table 1:** Clinical characteristics of study patients, C-reactive protein value, eosinophil count and leucocyte count in the diagnostic classes of patients on admission to the intensive care unit

Parameter	Total (n = 177)	Negative group (n = 37)	SIRS (n = 20)	Infected group (n = 120)	*P *value*
Age (years)	42 ± 19	38 ± 20	35 ± 18	44 ± 18	0.077
Male gender (n (%))	101 (57)	19 (51)	12 (60)	70 (58)	0.726
Mc Cabe index (n (%))					0.578
Nonfatal disease	138 (78)	31 (84)	16 (80)	91 (76)	
Ultimately and rapidly fatal disease	39 (22)	6 (16)	4 (20)	29 (24)	
Acute Physiology and Chronic Health Evaluation II score	12 ± 7	7 ± 5	9 ± 5	13 ± 6	<0.001
Sequential Organ Failure Assessment score	3 (1 to 8)	0 (0 to 2)	1 (0 to 4)	3 (1 to 6)	0.002
ICU length of stay (days)	5 (3 to 10)	3 (2 to 5)	6 (2 to 10)	7 (4 to 11)	0.001
Sites of infection (n (%))					
Respiratory tract	72 (60)				
Urinary tract	25 (21)				
Meningitis	16 (13)				
Other	7 (6)				
ICU mortality (n (%))	58 (33)	3 (8)	5 (25)	50 (42)	<0.001
Leucocyte count (cells/mm^3^)	13,666 ± 7,497	11,305 ± 5,136	14,595 ± 6,399	14,169 ± 8,113	0.128
Eosinophil count (cells/mm^3^)	13 (0 to 83)	146 (54 to 250)	22 (13 to 85)	8 (0 to 36)	<0.001
C-reactive protein (mg/l)	84 (31 to 155)	19 (36 to 79)	59 (16 to 84)	108 (58 to 197)	<0.001

Data are expressed as median (interquartile range) or as mean ± standard deviation. *P values are from the chi-squared test, one-way analysis of variance, or the Kruskal-Wallis test to compare the differences between the three groups. ICU, intensive care unit; SIRS, systemic inflammatory response syndrome group.

Patients were classified as follows (Figure 1): negative group, 21% (n = 37); SIRS group, 11% (n = 20); sepsis group, 23% (n = 41); severe sepsis group, 31% (n = 55); and septic shock group, 14% (n = 24). Diagnoses in the negative group were acute poisoning (n = 30), scorpion envenomation (n = 3), acute ischemic stroke (n = 2), and hypercalcemia (n = 2). SIRS was caused by acute exacerbation of chronic obstructive pulmonary disease (n = 6), acute asthma (n = 4), diabetic ketoacidosis (n = 4), acute poisoning (n = 3), cardiogenic shock (n = 2), and gastrointestinal hemorrhage (n = 1).

Infections were microbiologically documented in 70 of 120 patients (58.3%); 60% had Gram-positive infection and 40% had Gram-negative infection. The major sources of infection were the respiratory tract (60%) and the urinary tract (21%).

### Diagnostic accuracy

**Figure 2:**
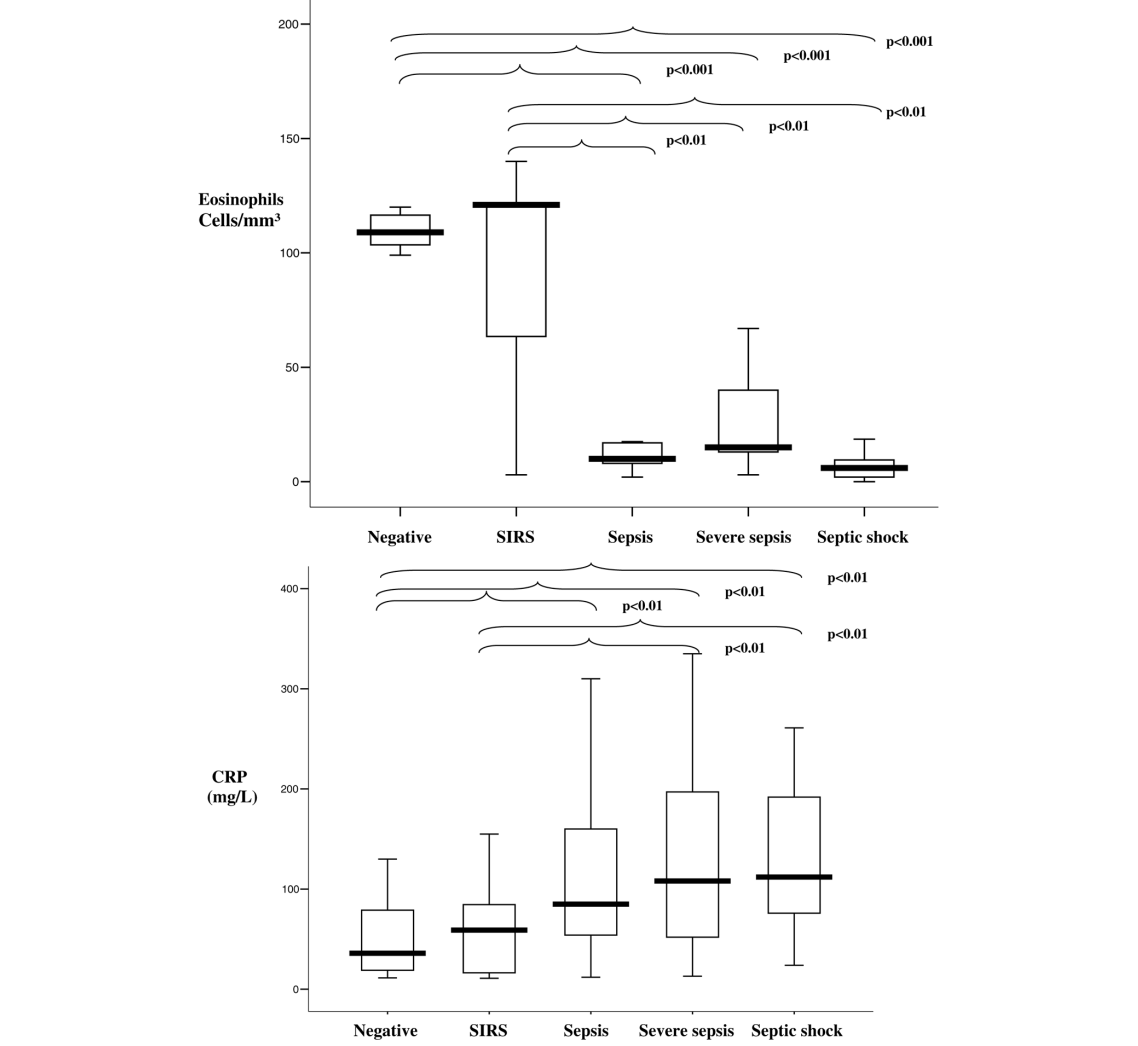
Eosinophil cell count and C-reactive protein level in the different diagnostic groups. Box plot of eosinophil cell count and C-reactive protein (CRP) level in the different diagnostic groups. SIRS, systemic inflammatory response syndrome. Central line, median; boxes, 25th to 75th percentiles; whiskers, 95% confidence intervals.

The comparison of the eosinophil cell count and CRP levels among the different groups showed significant differences (Kruskal-Wallis test, *P *< 0.001) (Figure 2). There were no differences in the leucocyte count between the different groups (one-way analysis of variance, *P *= 0.095).

**Figure 3:**
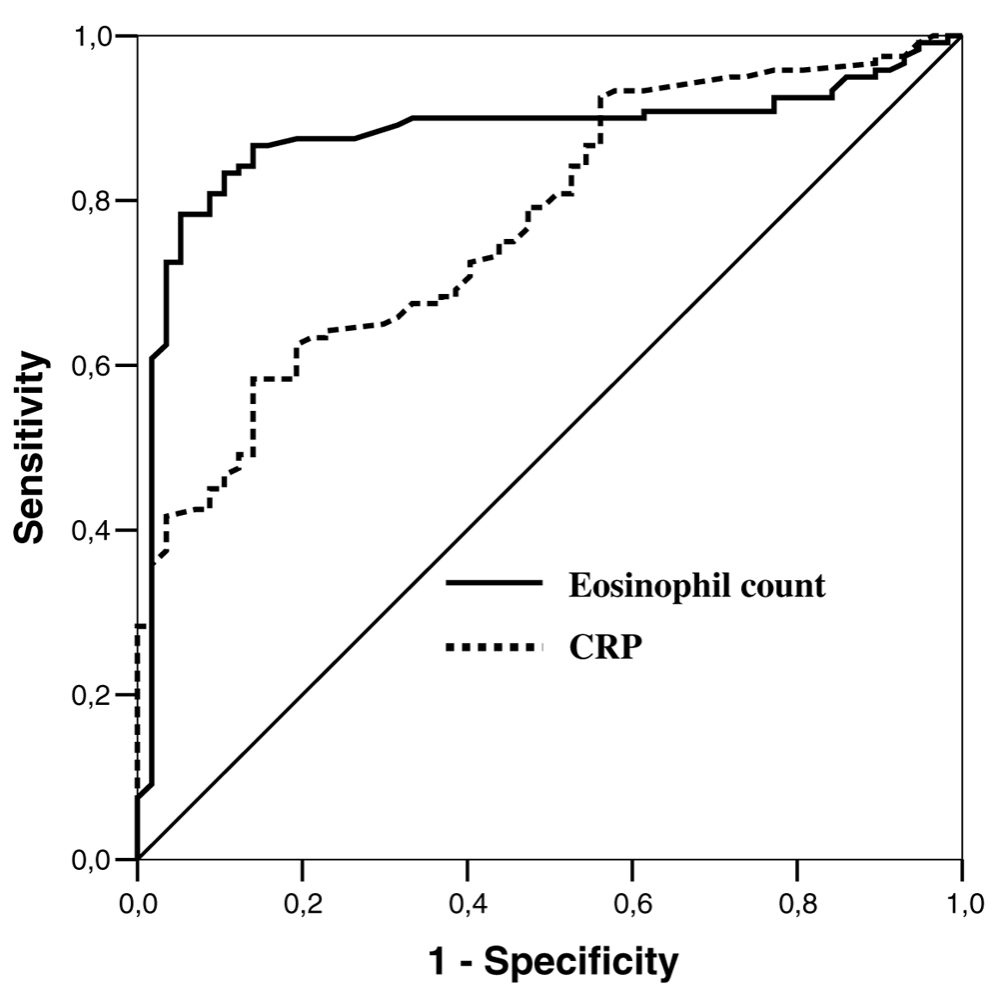
Eosinophil cell count and C-reactive protein level for discrimination of noninfection and infection. Receiver operating characteristic (ROC) curve of eosinophil cell count and C-reactive protein (CRP) level for the discrimination of noninfected patients (negative + systemic inflammatory response syndrome) and infected patients (sepsis + severe sepsis + septic shock). Areas under the ROC curves were 0.89 (95% confidence interval, 0.83 to 0.94) for eosinophils and 0.77 (95% confidence interval, 0.70 to 0.84) for CRP. Comparison of the areas under ROC curves between eosinophils and CRP, *P *= 0.010.

Concerning the comparison between the noninfected and infected groups, the median (interquartile range) eosinophil count was 109 (102 to 121) in noninfected patients and was 13 (8 to 28) in infected patients (*P *< 0.001). The median (interquartile range) CRP was 42 (18 to 79) and 108 (58 to 198) in the noninfected and infected groups, respectively (*P *< 0.001). Eosinophils had a higher discriminative value than the CRP level, with an area under the receiver operating characteristic curve of 0.89 (95% CI, 0.83 to 0.94) versus 0.77 (95% CI, 0.70 to 0.84) for CRP (*P *= 0.010) (Figure 3). At a cutoff value of 50 cells/mm^3^, eosinophils yielded a sensitivity of 80% (95% CI, 71% to 86%), a specificity of 91% (95% CI, 79% to 96%), a positive likelihood ratio of 9.12 (95% CI, 3.9 to 21), and a negative likelihood ratio of 0.21 (95% CI, 0.15 to 0.31) (Table 2). In multivariate logistic regression, the eosinophil cell count (adjusted odds ratio per 10-cell decrease, 1.09; 95% CI, 1.04 to 1.16; *P *= 0.002; frequency of significance in 1,000 bootstrap samples, 100%) and the CRP level (adjusted odds ratio per 1-point increase, 1.01; 95% CI, 1.00 to 1.01; *P*= 0.019; frequency of significance in 1,000 bootstrap samples, 98%) were found to be independent predictors of infection.

**Table 2:** Diagnostic performance of the eosinophil count and the C-reactive protein level in the prediction of sepsis on intensive care unit admission

Variable	Noninfection versus infection		SIRS versus infection	
	C-reactive protein level	Eosinophil cell count	C-reactive protein level	Eosinophil cell count

Cutoff value	>70 mg/l	<50 cells/mm^3^	>80 mg/l	<40 cells/mm^3^
Sensitivity (%)	68 (59 to 76)	80 (71 to 86)	68 (59 to 79)	80 (71 to 86)
Specificity (%)	61 (47 to 74)	91 (79 to 96)	55 (32 to 76)	80 (55 to 93)
Positive likelihood ratio	1.77 (1.25 to 2.51)	9.12 (3.9 to 21)	1.52 (0.92 to 2.50)	4.00 (1.65 to 9.65)
Negative likelihood ratio	0.52 (0.39 to 0.69)	0.21 (0.15 to 0.31)	0.57 (0.41 to 0.81)	0.25 (0.17 to 0.36)
Area under the receiver operating characteristic curve	0.77 (0.70 to 0.84)	0.89 (0.83 to 0.94)	0.77 (0.67 to 0.87)	0.84 (0.74 to 0.94)

Data in parentheses are 95% confidence intervals. Noninfection, negative + systemic inflammatory response syndrome (SIRS); infection, sepsis + severe sepsis + septic shock.

**Figure 4:**
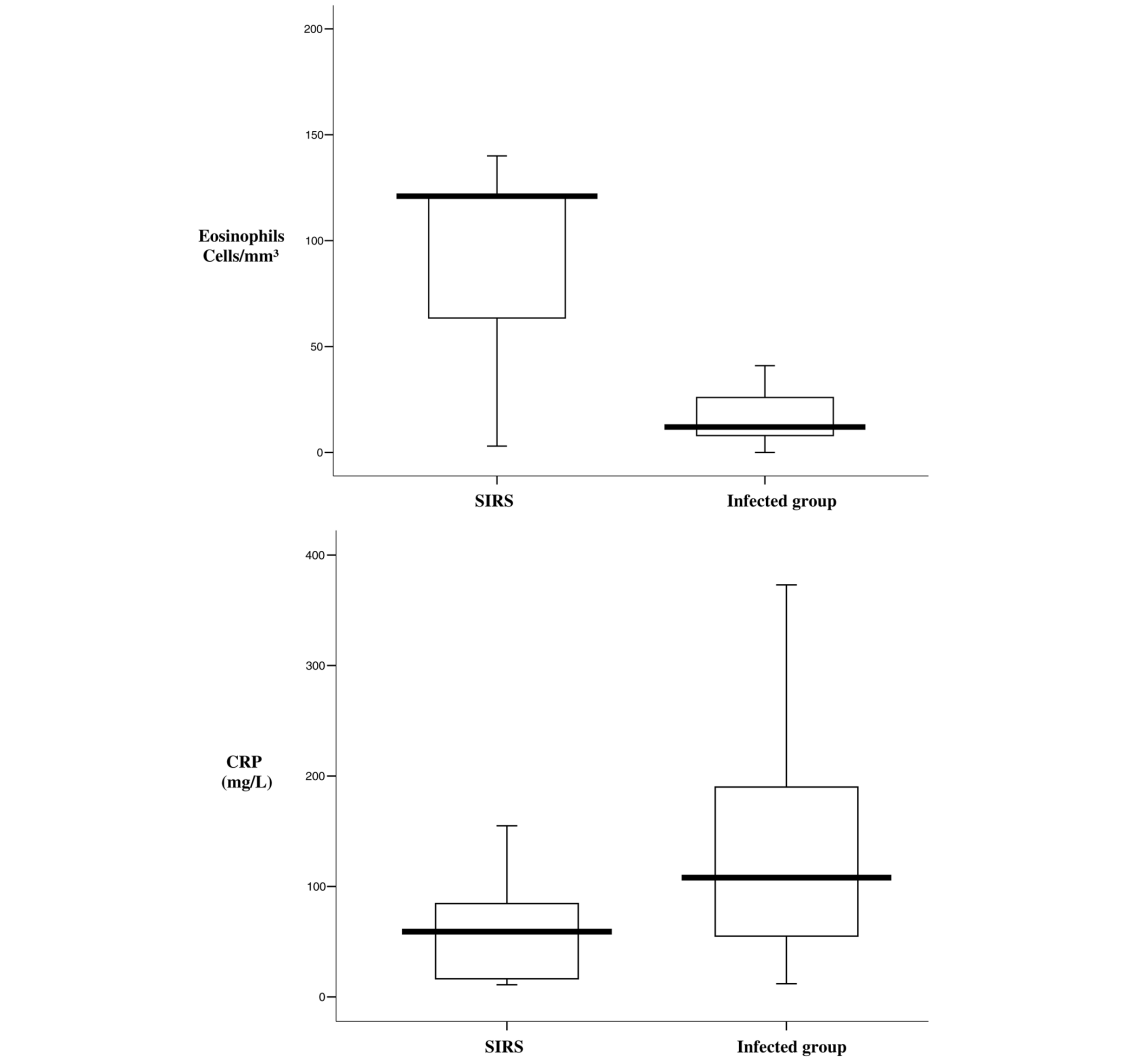
Eosinophil cell count and C-reactive protein level for comparison of systemic inflammatory response syndrome and infection (*P *< 0.001). Box plot of eosinophil count and C-reactive protein (CRP) level for comparisons between the systemic inflammatory response syndrome (SIRS) group and the infected group (sepsis + severe sepsis + septic shock). Central line, median; boxes, 25th to 75th percentiles; whiskers, 95% confidence intervals.*P *< 0.001 between eosinophils and CRP groups.

**Figure 5:**
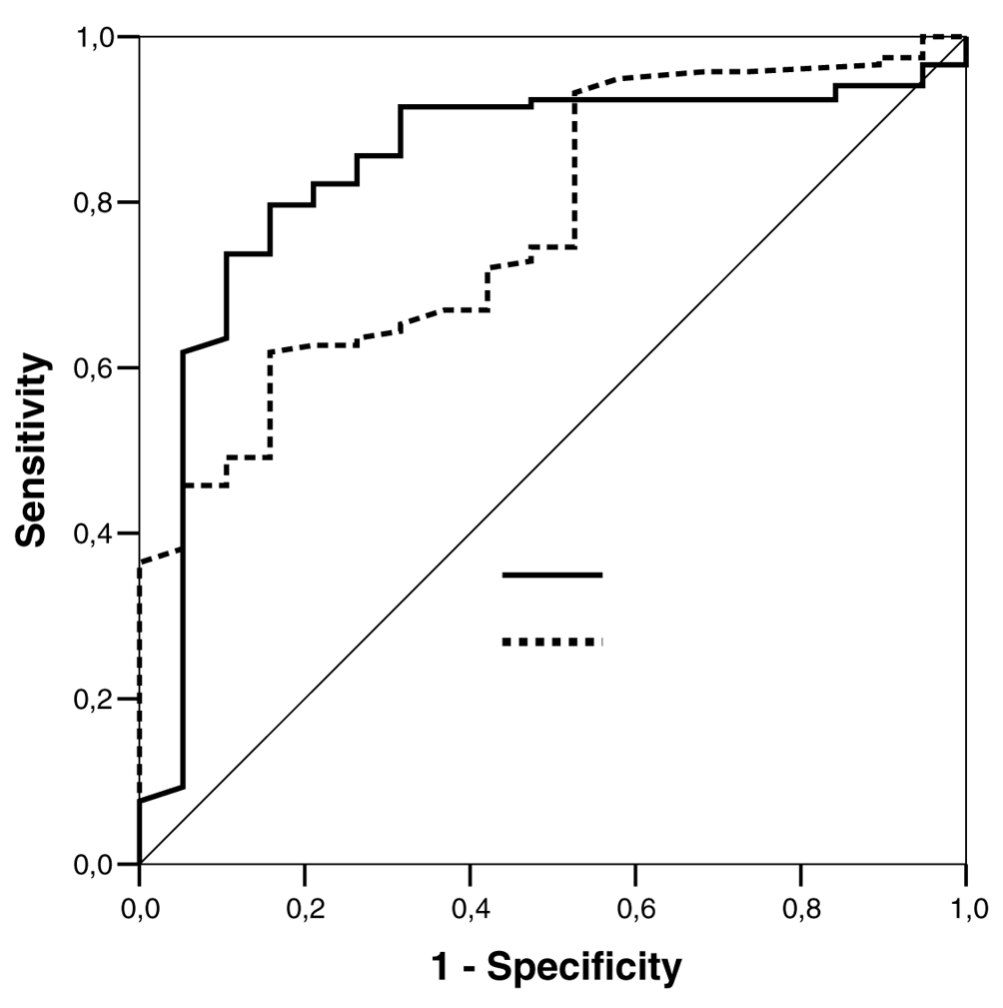
Eosinophil cell count and C-reactive protein level for discrimination of systemic inflammatory response syndrome and infection. Receiver operating characteristic (ROC) curve of eosinophil cell count and C-reactive protein (CRP) level for the discrimination of systemic inflammatory response syndrome patients and infected patients (sepsis + severe sepsis + septic shock). Areas under the ROC curves were 0.84 (95% confidence interval, 0.74 to 0.94) for eosinophils and 0.77 (95% confidence interval, 0.67 to 0.87) for CRP. Comparison of the areas under ROC curves between eosinophils and CRP, *P *= 0.175.

Concerning the comparisons between the SIRS and the infected groups (Figure 4), the median (interquartile range) eosinophil cell count was 121 (64 to 121) in SIRS patients and was 13 (8 to 28) in infected patients (*P *< 0.001). The median (interquartile range) CRP level was 59 (17 to 85) and 108 (58 to 198) in the SIRS and infected groups, respectively (*P *< 0.001). The area under the receiver operating characteristic curve was 0.84 (95% CI, 0.74 to 0.94) for eosinophils versus 0.77 (95% CI, 0.67 to 0.87) for CRP (Figure 5). The comparison of the areas under the receiver operating characteristic curves between eosinophils and CRP was not significant (*P *= 0.175). At a cutoff value of 40 cells/mm^3^, eosinophils yielded a sensitivity of 80% (95% CI, 71% to 86%), a specificity of 80% (95% CI, 55% to 93%), a positive likelihood ratio of 4 (95% CI, 1.65 to 9.65), and a negative likelihood ratio of 0.25 (95% CI, 0.17 to 0.36) (Table 2). In multivariate logistic regression, only the eosinophil cell count (adjusted odds ratio per 10-cell decrease, 1.07; 95% CI, 1.01 to 1.14; *P *= 0.019; frequency of significance in 1,000 bootstrap samples, 90%) was found to be an independent predictor of infection.

### Correlations

There were correlations between the eosinophil cell count and CRP level (*r *= -0.312, *P *< 0.001), between the eosinophil cell count and systolic blood pressure (*r *= 0.162, *P *= 0.030), between the eosinophil cell count and respiratory rate (*r *= -0.195, *P *= 0.011), between the eosinophil cell count and heart rate (*r *= -0.335, *P *< 0.001), and between the eosinophil cell count and Acute Physiology and Chronic Health Evaluation II score (*r *= -0.265, *P *< 0.001). No correlation was found between eosinophils and leucocytes or other clinical or biological features.

## Discussion

The present study is the first to suggest the value of eosinopenia in differentiating sepsis-related conditions from other inflammatory causes of SIRS in the ICU. Our results show the higher sensitivity and specificity of eosinopenia compared with the CRP level in the diagnosis of sepsis on admission to the ICU. Eosinopenia can therefore represent a good marker for the diagnosis of sepsis on ICU admission.

The diagnosis of sepsis is difficult, particularly in the ICU where signs of sepsis may be present in absence of a real infection [25]. The effort of many investigating groups has been to find a reliable marker to discriminate the inflammatory response to infection from other types of inflammation. Gold standards for the diagnosis of infection do not exist [3]; but procalcitonin is known to be among the most promising sepsis markers in critically ill patients, and is capable of complementing clinical signs and routine laboratory variables that are suggestive of sepsis [3-12]. The procalcitonin plasma concentration measure remains expensive in countries with low income and is not systematically used in our hospital. Our results, however, did show the interest of the cheap, old and forgotten sepsis marker that is eosinopenia, because it can perform as well as procalcitonin in the diagnosis of sepsis on ICU admission. Moreover, the effectiveness of this sepsis marker in our study was assessed in the differential diagnosis between all sepsis-related conditions and SIRS.

The mortality rate in our study seems high (33%) and is essentially related to infection. This observation can be explained as follows. First, infection is a common cause of admission to our medical ICU [26,27], which may be due to the lack of a specific unit for infectious diseases in our hospital, limited healthcare resources in the Moroccan context, delayed presentation of severely sick patients to the ICU, or a high prevalence of hospital-acquired infection in our hospital [28]. Second, our results show that mortality among the infected group was 42%; this rate appears to be high but is comparable with other studies [29,30] where reported ICU mortality related to sepsis conditions vary between 28% and 54%.

Eosinopenia was a sepsis marker in our study. The study by Gil and colleagues in a department of internal medicine showed that an inflammatory syndrome associated with an eosinophil count <40 cells/mm^3 ^is related to bacterial infectious diseases [19]. In an experimental study, Bass and colleagues produced eosinopenia in rabbits and in humans using chimiotactic factors of acute inflammation [14]. In trauma patients, however, Dipiro and colleagues found an increased eosinophil count after sepsis [31]. This eosinophil production was enhanced by IL-4 and IL-5, and suggests a T-helper lymphocyte type 2 cytokine activation in response to sepsis after traumatic injury.

Eosinophils normally account for only 1% to 3% of peripheral blood leucocytes, and the upper limit of the normal range is 350 cells/mm^3 ^blood [32]. The level of eosinophils in the body is normally tightly regulated. Mechanisms that control eosinopenia in acute infection, also considered as an acute stress, involve mediation by adrenal glucocorticosteroids and epinephrine [14]. Also, the initial eosinopenic response to acute infections was interpreted as being the result of a rapid peripheral sequestration of circulating eosinophils. Part of the sequestration could be ascribed to migration of eosinophils into the inflammatory site itself, presumably by chemotactic substances released during acute inflammation. The major chemotactic substances include C5a and fibrin fragments that have been detected in the circulation during acute inflammatory states [14].

If the hypothetical mechanism of eosinopenia as the migration of eosinophils to the inflammatory site is taken into account, this may explain the difference found between sepsis-related conditions and SIRS in our study. The lack of differences between sepsis, severe sepsis and septic shock groups, however, may be explained by the low rate of eosinophil count (near zero) in the infection groups. This suggests a floor effect of eosinopenia in infection groups.

The optimal eosinophil cutoff values have not yet been established and may differ depending on the clinical setting and the site and the etiology of infection. Furthermore, the diagnostic performance of eosinophils in our study is comparable with procalcitonin in patients with suspected sepsis in a study reported by Gibot and colleagues (sensitivity, 84%; specificity, 70%) [13]. In a meta-analysis the diagnostic performance of procalcitonin was low, with the mean value of both sensitivity and specificity being 71% (95% CI, 67 to 76) [33].

In the present study, eosinophils showed weak but significant correlations with sepsis parameters – and with the severity of the disease. In addition, eosinophils correlate to CRP, which is a proinflammatory marker. These correlations, although weak, seem to confirm the proinflammatory role of eosinophils in human sepsis. Gaïni and colleagues have also found weak correlation between the biological marker of infection high-mobility group-box 1 protein and proinflammatory markers [34]. Gibot and colleagues did not find any correlation between triggering receptor expressed on myeloid cells 1 and CRP, procalcitonin or any clinical and biological features [13].

The present study has a potentially important implication for clinicians in developing countries. As a cheap test to diagnose sepsis on ICU admission, eosinopenia offers a higher degree of certainty than other currently available tests or markers. Eosinopenia might guide physicians in their clinical decisions and may provide a stepwise approach to the complex management of critically ill patients.

The present study has several strengths. The study sample was large and involved a diverse group of critically ill adults admitted to a medical ICU. Blinded investigators determined each patient's diagnosis without knowledge of the eosinophil cell count and CRP levels. Finally, our study was designed as a real-life study. Some limitations of the study do, however, merit consideration. The eosinophil count and the CRP value were collected only on the day of ICU admission and not daily during the entire ICU stay. This cannot allow us to generalize our findings to evaluate the prognostic value of eosinopenia. As we used clinical criteria and microbiological evidence, it may have been difficult to ascertain the exact cause of SIRS in all patients. No surgical patients were enrolled because of the specificity of our medical ICU. Finally, infections were microbiologically documented in 58% of cases; this low percentage may be explained by the frequency of respiratory tract infection in our study (60% of cases), because microorganisms are not usually isolated in respiratory tract infections [35,36].

## Conclusion

Eosinopenia can be used as a diagnostic marker of sepsis in newly admitted critically ill patients. Eosinopenia is a better diagnostic marker than CRP, and may become a helpful clinical tool in ICU practices. Further studies are needed to evaluate the progression of eosinopenia with the severity of sepsis and to establish the best cutoff values for this marker.

## Key messages

• The present study is the first report testing the value of eosinopenia in the diagnosis of sepsis on admission to the ICU.

• Eosinopenia is a good diagnostic marker in distinguishing between noninfection and infection in newly admitted critically ill patients.

• Eosinopenia is a moderate marker in discriminating between SIRS and infection in newly admitted critically ill patients.

• Eosinopenia showed a higher sensitivity and specificity compared with CRP in the diagnosis of sepsis on admission to the ICU

• Eosinopenia may become a helpful clinical tool in ICU practices.

## Abbreviations

CI = confidence interval; CRP = C-reactive protein; ICU = intensive care unit; IL = interleukin; PCR = polymerase chain reaction; SIRS = systemic inflammatory response syndrome.

## Competing interests

The authors declare that they have no competing interests.

## Authors' contributions

KA and IK contributed equally to the work. KA and IK drafted the manuscript and participated in the acquisition of data and the study design. JB participated in the acquisition of data. NM helped to draft the manuscript, and participated in the acquisition of data. AZ participated in the coordination of the study. AAZ participated in the design of the study, and performed the statistical analysis. RA conceived of the study, participated in the design of the study, performed the statistical analysis and interpretation of data, and gave the final approval of the manuscript. All authors read and approved the final manuscript
